# Oxytocin quality: evidence to support updated global recommendations on oxytocin for postpartum hemorrhage

**DOI:** 10.1186/s40545-020-00205-7

**Published:** 2020-05-15

**Authors:** Peter Lambert, Michelle P McIntosh, Mariana Widmer, Lawrence Evans, Megan Rauscher, Rutendo Kuwana, Fiona Theunissen, Beth Yeager, Helen Petach

**Affiliations:** 1 Drug Delivery Disposition and Dynamics, Monash Institute of Pharmaceutical Sciences, Monash University, Parkville, Australia; 2 Department of Reproductive Health and Research, World Health Organization, Geneva, Switzerland; 3 Promoting Quality of Medicines Plus Program, U.S. Pharmacopeial Convention, Rockville, MD USA; 4 Global Health Supply Chain Program-Procurement and Management Project, Chemonics International, 251 18th Street South, Suite 1200, Arlington, VA 22202 USA; 5 Regulatory Systems Support, World Health Organization, Geneva, Switzerland; 6 Independent Consultant, Geneva, Switzerland; 7 Office of Maternal and Child Health and Nutrition, Bureau for Global Health, United States Agency for International Development, Washington, DC USA

## Abstract

**Background:**

The use of quality injectable oxytocin effectively prevents and treats postpartum hemorrhage, the leading cause of maternal death worldwide. In low- and middle-income countries (LMICs), characteristics of oxytocin—specifically its heat sensitivity—challenge efforts to ensure its quality throughout the health supply chain. In 2019, WHO, UNFPA and UNICEF released a joint-statement to clarify and recommend that oxytocin should be kept in the cold chain (between 2 and 8 °C) during transportation and storage; however, confusion among stakeholders in LMICs persists.

**Objectives and methods:**

To further support recommendations in the WHO/UNFPA/UNICEF joint-statement, this paper reviews results of oxytocin quality testing in LMICs, evaluates product stability considerations for its management and considers quality risks for oxytocin injection throughout the health supply chain. This paper concludes with a set of recommended actions to address the challenges in maintaining quality for a heat sensitive pharmaceutical product.

**Results:**

Due to the heat sensitivity of oxytocin, its quality may be degraded at numerous points along the health supply chain including:

- At the point of manufacture, due to poor quality active pharmaceutical ingredients; lack of sterile manufacturing environments; or low-quality manufacturing processes
- During storage and distribution, due to lack of temperature control in the supply chain, including cold chain at the end user health facility

Safeguarding the quality of oxytocin falls under the purview of national medicines regulatory authorities; however, regulators in LMICs may not adhere to good regulatory practices.

**Conclusions:**

Storing oxytocin from 2 to 8 °C throughout the supply chain is important for maintaining its quality. While short temperature excursions may not harm product quality, the cumulative heat exposure is generally not tracked and leads to degradation. National and sub-national policies must prioritize procurement of quality oxytocin and require its appropriate storage and management.

## Introduction

Postpartum hemorrhage (PPH)–or excessive bleeding after childbirth–is the leading cause of maternal mortality worldwide [1]. The World Health Organization (WHO) estimates that approximately 303,000 maternal deaths occur every year with nearly 20% of those resulting from PPH [1]. PPH is both preventable and treatable with inexpensive medicines that are widely available [2]. Oxytocin injection is still a preferred medicine for prevention and treatment of PPH. According to WHO recommendations, oxytocin (10 international units [IU], intramuscular [IM]/intravenous [IV]) is the recommended uterotonic agent for the prevention of PPH for all births in all settings unless oxytocin is unavailable or its quality cannot be guaranteed [3]. If bleeding occurs, additional units (up to 40 IUs total) should be administered intravenously until bleeding stops [4]. Recent innovations and updates to WHO recommendations have increased the range of options for prevention and treatment of PPH. In the new recommendations, heat stable carbetocin is included for the prevention of PPH [3]. However, at the time of writing, heat-stable carbetocin is not yet available in most LMICs and is only indicated for the prevention of PPH. LMICs will need to ensure that high quality oxytocin is available for years to come, including in other indications such as induction of labor, augmentation of labor or treatment of PPH.

The effectiveness of oxytocin is widely recognized [4, 5]; however, quality oxytocin is not always available in LMICs due quality deficiencies at the point of manufacturer and excessive exposure to higher than acceptable temperatures. In 2019, WHO, the United Nations Population Fund (UNFPA), and the United Nations International Children’s Fund (UNICEF) released a joint-statement that clarifies storage requirements for oxytocin, stating that oxytocin should be labeled for storage between 2 and 8°Celsius (C) and managed in a cold chain of 2–8 °C for storage and distribution [6]. This statement clearly states that oxytocin should remain in cold storage throughout the supply chain; however, there are still oxytocin products with differing storage conditions on their labels, including for storage < 25° or < 30° C [7]. This inconsistency creates confusion among stakeholders in LMICs continues, and oxytocin is often stored and transported in hot, ambient conditions. Use of poor-quality oxytocin may result in additional interventions, including administration of additional uterotonics to prevent PPH and a range of surgical, medical and manual interventions to treat PPH. These interventions are required to treat hemorrhage and prevent mortality.

## Objectives and methods

This paper expands upon and further supports the WHO/UNFPA/UNICEF recommendations on management of oxytocin throughout the health supply chain. In October 2017, an expert technical advisory group (TAG) convened to review existing data on oxytocin quality and issues that challenge efforts to maintain the product’s quality. Based on available information, the TAG agreed on oxytocin-related quality challenges that occur throughout the health supply chain. Following the meeting, peer-reviewed and grey literature sources were reviewed for each paper sub-topic to ensure alignment with broader research and evidence. Searches were performed in PubMed and Google using combinations of general terms, including “oxytocin”, “quality” and “hemorrhage”. Citations and references were reviewed, relevant papers were further examined, and supplemental targeted searches were used to identify new and pertinent articles and reports. Reviewer bias was addressed during the initial TAG meeting and during subsequent feedback reviews. The synthesis of findings is described in Sections 3–5. This paper includes a set of recommended actions to address the challenges in maintaining quality for a heat-sensitive pharmaceutical product (Section 6).

## High prevalence of poor-quality oxytocin in LMICs

Poor quality oxytocin is prevalent in LMICs and is well-documented [8]. Oxytocin injection has specific characteristics—namely, that all formulations are heat-sensitive—that challenge efforts to ensure its quality throughout the health supply chain. Excessive heat exposure can lead to a reduction in content of active pharmaceutical ingredients (APIs) in oxytocin products along with a commensurate increase in degradation products, for which there is limited safety information. Furthermore, manufacturing deficiencies can lead to variable amounts of API in the finished product as well as contaminants and/or non-sterile products.

A 2016 systematic review reported widespread oxytocin that did not meet quality assurance standards [8]. The review found that of 559 samples of oxytocin, collected non-systematically across 15 LMICs, unacceptable oxytocin content was found in 36% of tested samples based on International Pharmacopeia requirements (no less than 90% and no more than 110%) [8]. The highest proportion of samples with inadequate API amounts were from Africa (60% of the total tested failed analysis) [8]. These test failures highlight that there is a measurable amount of poor-quality oxytocin injection in circulation. However, it is important to recognize that the studies included in this review did not sample representatively or randomly, and it is unlikely that 60% of all oxytocin in Africa is poor-quality. The review also included samples collected from both central medical stores and facility level settings, thus, it is not clear where product quality was compromised (at the point of manufacture or in the supply chain). Table 1 provides a summary of oxytocin quality results, described in the 2016 systematic review.

**Table 1:** Select results on oxytocin quality in LMICs

Author, Year	Location	Type of Publication	Sample Size	Selected Results
Lambert et al., 2018 [8]	Democratic Republic of the Congo	Peer-reviewed article	Where available, 20 ampoules from 15 facilities (256 ampoules tested in total)	80% out specification
Anyakora et al., 2018 [9]	Nigeria	Peer-reviewed article	159 ampoules tested	74.2% out of specification
National Institute of Biologics, Ministry of Health and Family Affairs of India, 2017 [10]	India	Government report	58 samples drawn from government sources	41.3% not of standard quality
M. Lui et al., 2016 [11]	Nepal, Vietnam	Peer-reviewed article	42 samples from 35 pharmacies	31% out of specification
Torloni et al., 2016 [7]	15 countries	Peer-reviewed article (systematic literature review)	8 studies assayed a total of 559 oxytocin samples	45.6% median prevalence of oxytocin samples did not pass quality testing as defined by authors; 36.0% out of specification
World Health Organization, 2015 [12]	Burkina Faso, Kenya, Madagascar, Nepal, Nigeria, Tajikistan, Tanzania, Vietnam, Zimbabwe	WHO report	22 batches from 15 manufacturers	64% out of specification
Stanton et al., 2014 [13]	India	Peer-reviewed article	193 ampoules	36.2% out of specification
Ghana Food and Drugs Authority (FDA) Laboratory Services Department, 2013 [14]	Ghana	Government report	169 ampoules	Assay: 55.6% out of specification (2% of these had 0% API); 10% failed assay but passed sterility Sterility: 40% of samples failed sterility but passed assay; 45% of samples failed both assay and sterility tests 97.5% of samples failed sterility, assay, or both
Stanton et al., 2012 [15]	Ghana	Peer-reviewed	46 ampoules	73.9% out of specification; 4.3% expired
Hogerzeil et al., 1993 [16]	Zimbabwe	Peer-reviewed	6 samples from the same manufacturer from five district hospitals	1 of 6 samples expired; of remaining 5 samples, 80% out of specification

While a direct correlation between the prevalence of poor quality of oxytocin and high mortality rates due to PPH is yet to be formally established, there is evidence for clinical consequences resulting from poor-quality oxytocin at the point-of-use. For example, in a Myanmar qualitative study, where oxytocin is not routinely stored or transported in the cold chain (contrary to WHO recommendations), obstetricians and midwives report that both high doses of oxytocin and routine, concurrent administration of misoprostol are required to effectively prevent and treat PPH [17]. This study suggests that the oxytocin doses may not contain the expected amounts of API.

## Testing and labeling requirements

National medicines regulatory authorities typically require that medicines be stability-tested in the same conditions as those expected for storage, and those test conditions are then reflected on the product labeling. For example, a 2 year stability study was required to establish guidelines for the storage of ergometrine, methylergometrine and oxytocin in tropical climates [18]. In this same study, oxytocin API levels fell below the industry-specified standard of 90% during the 2 years when exposed to temperatures above 30 °C [18]. Based on these findings, the study authors recommended that oxytocin be labelled with “keep under refrigeration” [18].

### Manufacturer stability tests

Recommended storage conditions on product labels are based on manufacturer tests of stability and should reflect the temperatures expected in the intended country of use. According to WHO guidelines, manufacturer-recommended storage conditions and associated labeling for pharmaceutical products for country distribution should reflect the country climatic conditions [19, 20]. The manufacturers must test pharmaceutical products and determine the storage conditions that ensure long-term product stability in the local climate [21]. While WHO provides guidance and specifications, it is the responsibility of the manufacturer to carry out product testing and ensure appropriate product labelling during product registration. These requirements are applicable to all products, but are particularly pertinent to heat-labile products, such as oxytocin. And, the onus of product testing falls to the manufacturer.

Manufacturers may become discouraged by costly product testing combined with long regulatory submission and approval processes [22], and this may deter manufacturers from entering many LMIC markets. To reduce costs, product testing–including that for oxytocin–may be limited to a narrow range of climatic conditions. The data generated from product testing may then be insufficient to support the management of the product outside of those conditions such as a country with higher ambient air temperature.

**Fig. 1:**
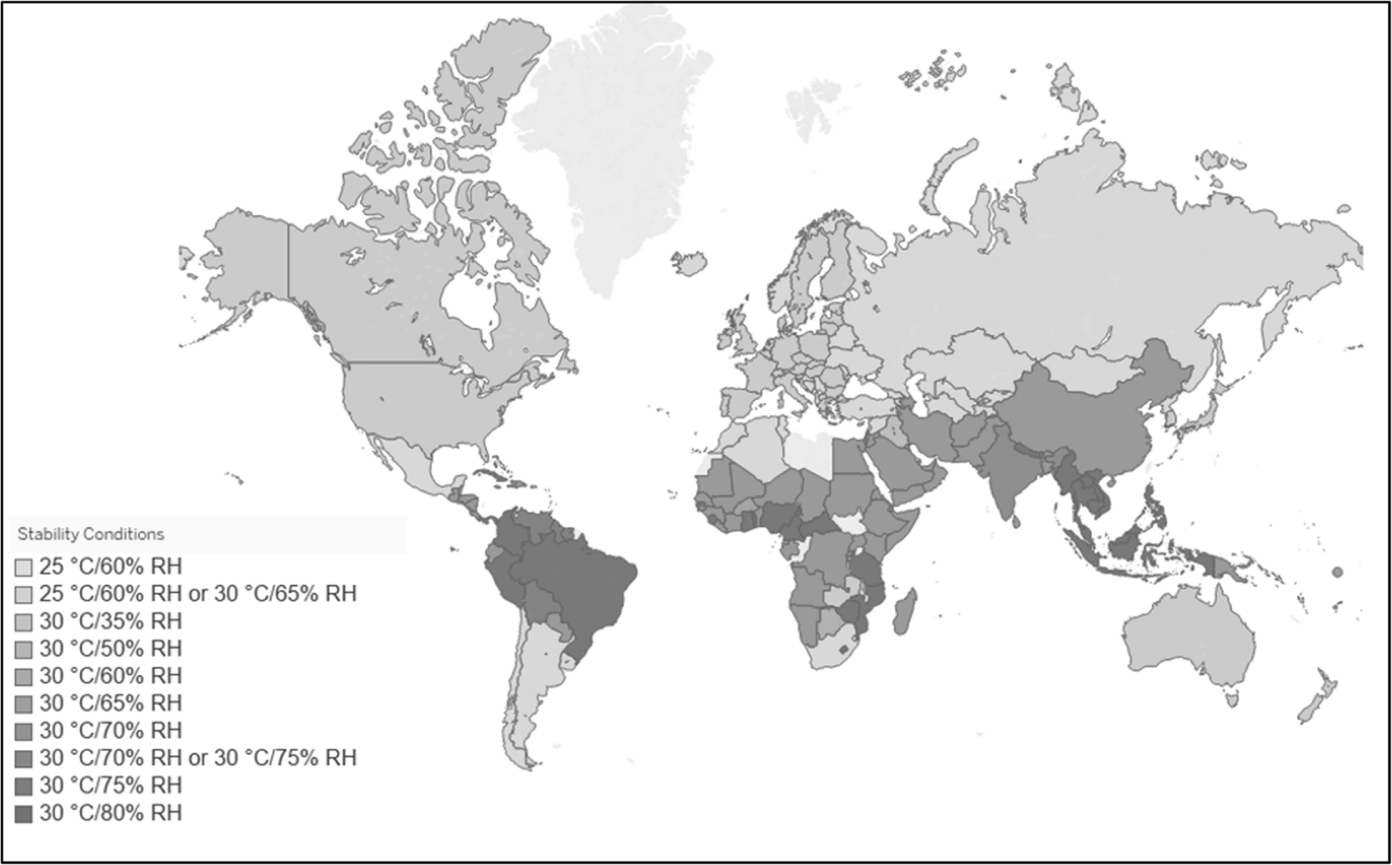
Map of WHO Member States and the Required Temperature and Relative Humidity Conditions for Stability Testing of Non-Refrigerated Pharmaceuticals [14] In 2015, WHO developed an updated list of WHO member states and the temperature and relative humidity conditions at which stability testing of pharmaceutical products must be conducted to allow non-refrigerated storage in that country, as illustrated in the map (above)

In countries with high ambient temperatures and/or high relative humidity (designated as climatic zones III and IV by WHO guidelines), manufacturer testing must demonstrate product stability at 30 °C for the duration of shelf-life if a product is labelled for storage outside of the refrigerator [19, 23]. As indicated previously, oxytocin injection is an example of a product that is not adequately stable at this temperature [18]. Therefore, oxytocin injection products that enter the market in these climatic zones III and IV countries (see Fig. 1) should be labelled for refrigerated storage at 2–8 °C throughout the shelf-life of the product.

**Fig. 2:**
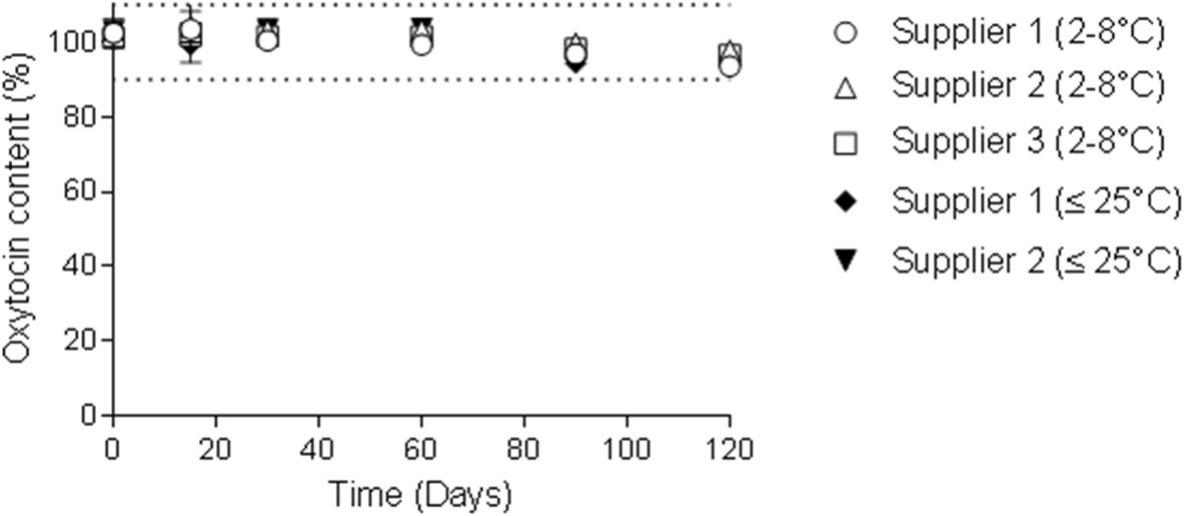
Stability study comparing content of oxytocin 10 IU/mL injection ampoules labelled for storage at < 25 °C with those labelled for storage at 2–8 °C when stored at 30 °C for 120 days [24]

Currently, several oxytocin injection products are marketed in climatic zones with temperatures that exceed 25 °C, yet their product labels only recommend storage at < 25 °C or similar (e.g., store in a cool place or at room temperature). The products labeled for storage at < 25 °C are no more stable than those labeled for storage at temperatures between 2 and 8 °C [24]. Independent testing by the United Nations Population Fund (UNFPA) and Monash University compared the stability of products labeled for storage 2–8 °C and compared with products labeled for storage < 25 °C and showed similar degradation profiles across a wide range of temperatures. The variety of storage temperatures on the product labels do not correspond to different product stabilities. See Fig. 2 for data of storage at 30 °C [24].

### Pharmacopeia specifications and test procedures

Pharmacopeia monographs contain specifications and testing procedures that serve as the foundation of standards and quality specifications for API and finished pharmaceutical products (FPP). They include detailed information on testing for identification, purity and unwanted impurities, all of which may inform the manufacturing process or product stability.

The different pharmacopeias have different descriptions for product testing and storage conditions. In the oxytocin API monographs for five of the six pharmacopeias (*British Pharmacopeia, European Pharmacopeia, Indian Pharmacopeia, International Pharmacopeia, Japanese Pharmacopeia and United States Pharmacopeia*), storage conditions for 2-8 °C or “preserve in refrigerator” can be found. Only the *International* and *Japanese Pharmacopeias* present similar storage conditions for oxytocin injection, even given its known stability issues. Only the *International Pharmacopeia* has a procedure to assess the stability of oxytocin injection by examining both the API and any resulting impurities. Table 2 summarizes select quality specifications for oxytocin API and FPP [7]. Additional specifications and procedures can be found in the respective pharmacopeia monographs. These monographs are critical to ensuring the uniform specification of product quality.

**Table 2:** Select pharmacopeia monograph quality specifications for oxytocin API and FPP

Pharmacopeia	Storage conditions		Assay		Related substances/Impurities	
	API	FPP	API	FPP	API	FPP
*United States Pharmacopeia* 40	Refrigerated, 2-8 °C	No specification provided	NLT 90.0 and NMT 110.0%	NLT 400 USP Oxytocin units per mg	Total impurities NMT 5%	No specification provided
*European Pharmacopeia*, 9th Ed	2-8 °C, protect from light	NM	NLT 93.0% and NMT 103.0%	NM	Any individual impurity NMT 1.5%;	NM
					Total impurities NMT 5%	
*International Pharmacopeia*, 2017, 7th Ed	2-8 °C, protect from light	Unless otherwise indicated on the label, 2-8 °C, The manufacturer may provide additional information on the label regarding storage conditions for a specified period which may differ from the long-term storage conditions.	NLT 93.0% and NMT 103.0%	NLT 90.0% and NMT 110.0%	Any individual impurity NMT 1.5%;	Any individual impurity NMT 1.5%;
					Total impurities NMT 5%	Total impurities NMT 5%
*British Pharmacopeia*, 2018	2-8 °C, protect from light	No specification provided	NLT 93.0% and NMT 103.0%	NLT 90.0% and NMT 110.0%	Any individual impurity NMT 1.5%;	No specification provided
					Total impurities NMT 5%	
*Japanese Pharmacopeia*, 2016, 17th Ed,	2-8 °C,	In a cold place (1-15 °C), avoid freezing	NLT 540 oxytocin Units and NMT 600 oxytocin Units	NLT 90.0% and NMT 110.0%	Any individual impurity NMT 1.5%;	No specification provided
					Total impurities NMT 5%	
*Indian Pharmacopeia* 2014	Store protected from moisture	No storage requirements provided	NLT 95.0% and NMT 105.0%	NLT 90.0% and NMT 110.0%	No specification provided	No specification provided

*NLT* not less than, *NMT* not more than, *IU* international unit, *NM* No monograph

## Oxytocin quality issues exist throughout the health supply chain

**Fig. 3:**
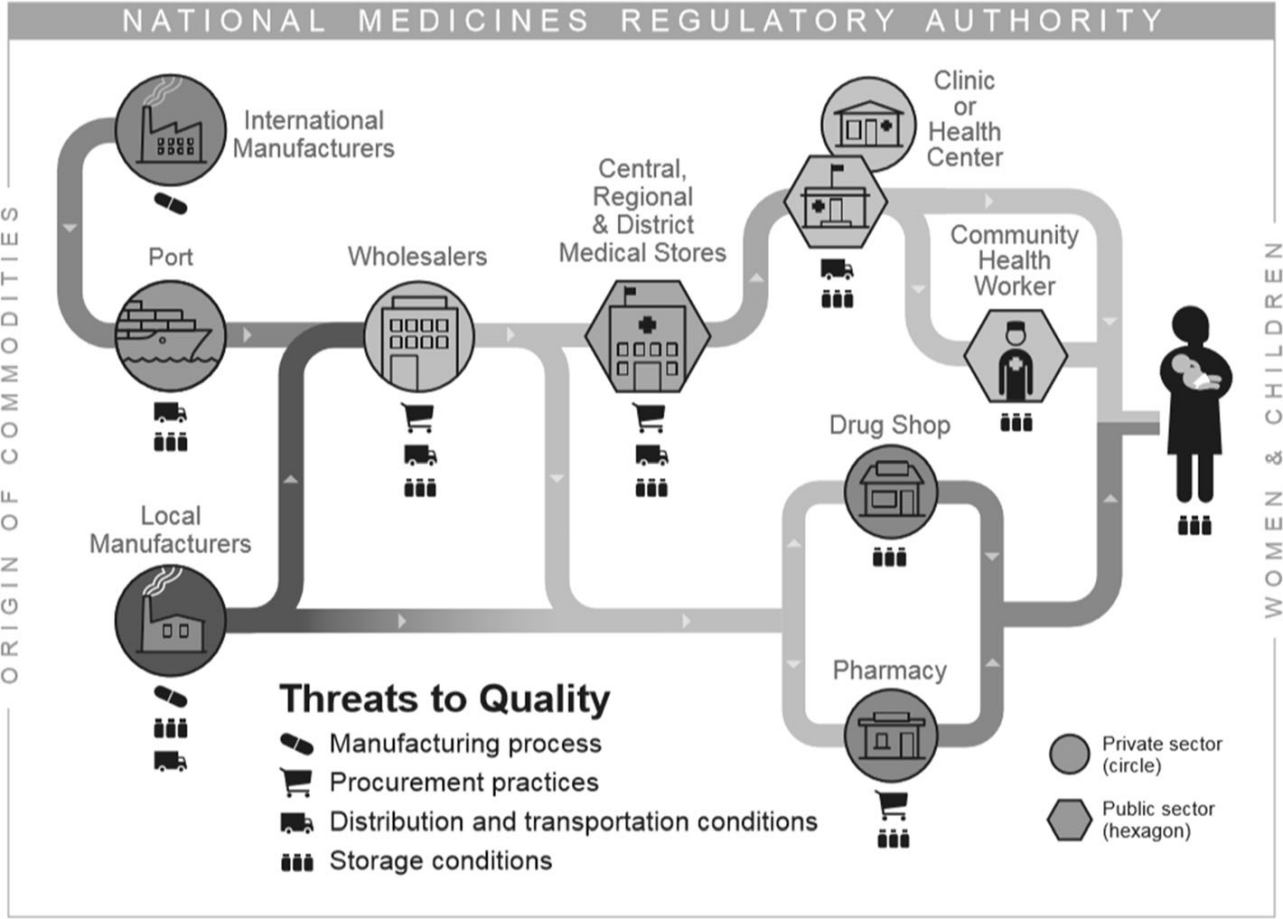
Threats to Oxytocin Quality Occur throughout the Supply Chain for Oxytocin. Threats to oxytocin quality occur throughout manufacturing, procurement, distribution, and storage

The health supply chain is complex and includes numerous steps where temperature excursions may occur and require special handling for oxytocin. Some susceptible points along the supply chain include shipping from the manufacturer, storage at the port of importation, transportation across the country, and ongoing transportation and storage throughout the supply chain (including medical warehouses, health centers, and end user facilities). Figure 3 depicts an illustrative flow of commodities throughout the country-level health supply chain and the points where commodities, such as oxytocin, may be exposed to quality threats. In the case of oxytocin, even if quality product is manufactured, the transportation and storage conditions throughout any location in the supply chain may compromise the quality of the medicine.

### Manufacturing processes

Oxytocin quality issues may begin at the point of manufacture. Manufacture of oxytocin injection is a two-part process: 1) manufacture of the API and 2) transformation of the API into the FPP, in which the API is mixed with excipients (e.g., saline) and packaged in a sterile environment. Many oxytocin injections available in LMICs are produced in China, India and Indonesia by FPP manufacturers [8, 25], with most API originating in China or India.

Since the API used for manufacturing oxytocin injections is an important determinant of the quality of the final product, WHO indicates that FPP manufacturers should ensure that production includes quality API [26]. The quality assurance of API may be verified at several levels:

- Certification by the authorities in the country of manufacture of compliance with Good Manufacturing Practices (GMP)
- Certification of the FPP by a regulatory authority with the capability of validating the full technical dossier of the FPP
- Certification of the processes of supplier qualification by the FPP manufacturer as part of their GMP processes

To date, the WHO Prequalification Program has not yet listed any oxytocin APIs, but there are two prequalified FPPs. The listing of FPPs infers acceptable quality API through the prequalification of the full technical dossier of the FPPs [27].

Manufacturers are often challenged by needing to meet international quality standards. There are a limited number of companies that are capable of manufacturing high-quality generic products that can provide a complete registration dossier for submission to the national medicines regulatory authority outside their home markets [28]. As a comparison from contraceptive market, 44 contraceptive manufacturers from 15 countries received national GMP certification, but less than 30% would be able to meet the current GMP requirements of WHO, the Pharmaceutical Inspection Cooperation Scheme, or any stringent regulatory authority (SRA) [28]. This study illustrates that in numerous examples, authorization at the national level is not equivalent to authorization by an SRA. Few manufacturers will be able to meet the stringent requirements of an SRA.

### Procurement practices

Procurement is one of the supply chain activities where product quality can be demanded. Oxytocin is procured throughout the health supply chain, including through procurement agents at the central medical stores, state-level equivalents, and health care providers. Procurement can also occur at public and private hospitals and health centers and can include sourcing of oxytocin through the public supply chain and through private sector wholesalers and suppliers. Rigorous procurement practices—including requirements for the procurement of SRA approved or WHO prequalification or other relevant approvals—help ensure the quality of oxytocin products in LMICs. Conversely, procurement practices that do not prioritize quality can negatively affect the quality of oxytocin available to the end user. Rigorous procurement is only one part of a quality supply chain; additional regulatory oversight is necessary for post-market surveillance and ensuring that only quality products are sourced into the supply chain by public and private sector suppliers.

High-quality regulation supports quality procurement by ensuring that only registered products can enter the country [8]. WHO Operational Principles for Good Pharmaceutical Procurement (1999) recommend formal supplier prequalification and monitoring, transparency and written procedures, separation of functions, and a product quality assurance program as key elements of a procurement system that guarantees product quality [29].

Procurement practices should never prioritize price over quality product. Nearly 300 different oxytocin products are produced by more than 100 manufacturers [2], resulting in a highly competitive market where manufacturers may compromise quality to achieve lower costs and increased sales. National policies—such as those that regulate pharmaceutical prices and reimbursement rates—may increase pressure to reduce the price of oxytocin. There is evidence of extreme price pressure in India where national regulations, i.e., the Drugs Price Control Order, limit prices for essential medicines, and states compete to further reduce medicine prices. Prices for oxytocin injection have been found as low as 1.2 Indian rupees for 10 IU of oxytocin (approximately USD $0.02) [30]. Similarly, in Ghana, national health insurance reimbursement rates for 10 IUs of oxytocin are approximately USD $0.04 [31]. However, SRA-approved or prequalified oxytocin injection is typically priced above this level, partly due to the cost recovery required for quality oxytocin production [30]. The median price of 10 IUs of oxytocin, which likely includes a combination of with and without quality assurance, was US $0.15 in 2015 according to the International Medical Products Price Guide [32].

### Distribution, transportation and storage conditions

The entire supply chain must collectively maintain the integrity of appropriate storage of oxytocin, including national or regional warehouses, health facilities, and distribution systems [33, 34]. WHO recommends temperature monitoring and mapping during distribution and storage of essential medicines [33]; in practice, temperature excursions are known to occur, particularly in LMICs with high ambient temperatures where resources for temperature-controlled storage infrastructure and monitoring equipment are lacking [7]. A 2017 study monitored storage temperatures of oxytocin injection supplies from a European manufacturer throughout the supply chain to facilities in Ghana [35]. Recorded temperatures ranged from − 9.9 °C to + 30 °C [35]. Similarly, high temperature conditions have been reported along supply chains to other LMICs in hot countries [18]. This indicates that oxytocin supplies are at real risk of exposure to extreme temperatures and potential degradation during storage, transport and distribution in these territories [35, 36].

Surveys have identified examples of oxytocin temperature excursions and the factors that lead to those excursions. Oxytocin storage conditions at the first level of country distribution were measured during a 2015 maternal, newborn and child health commodities survey [7]. Results from 10 countries revealed that only nine out of 21 samples were stored as per the manufacturers’ instructions on the day the sample was collected, suggesting supply chain managers may not consistently monitor the storage temperature of oxytocin [7]. Additionally, labels of 10 samples permitted storage outside of the refrigerator, despite nine of 10 source countries being in climatic zones III and IV [7]. Where procured oxytocin is incorrectly labeled for the climate of that country (see Testing and labeling requirements), the supply chain managers may effectively monitor storage, but against inappropriate temperature limits. Similar deficiencies can occur at the point of use. Multiple reports detail circumstances where oxytocin is stored outside of appropriate cold chain conditions, especially in facilities at lower levels of the health system that do not have appropriately designed cold chain infrastructure [17, 37]. These deficiencies occur both in the storage area of facilities and in the labor room where ambient temperatures can be high [37].

Inadequate personnel training and inadequate facilities may also contribute to inadequate storage and management of oxytocin. A qualitative study in Ethiopia, India and Myanmar found that oxytocin storage knowledge is highly variable among clinicians, particularly in India and Myanmar, as they showed poor understanding of cold chain storage requirements for oxytocin [17]. Myanmar clinicians were aware that cold chain infrastructure earlier in the supply chain was lacking and consequently recognized little value in attempting to maintain refrigerated oxytocin storage at facility level [17].

The complexity of the supply chain leads to numerous different reasons for oxytocin storage outside of the cold chain.

### Recommended dose

Another complexity for oxytocin in the supply chain is the inclusion of multiple doses that may lead to inappropriate treatment. WHO recommends the use of 10 IU of oxytocin (IV or IM) for prevention of PPH, with intramuscular administration recommended for use in resource poor settings [3, 4, 38]. While a 10 IU oxytocin dose is recommended for PPH, both 10 IU/mL and 5 IU/mL ampoules are commonly available from manufacturers to accommodate the use of oxytocin injection for other indications where lower doses are required (i.e., induction and augmentation of labor). No research study has directly investigated the difference between 5 IU and 10 IU for prevention and treatment of PPH, however, there are suggestions that 5 IU may be less effective when used intramuscularly. In an early study of the prevention of PPH using oxytocin, dosing started at 5 IU IM, but had to be increased to 10 IU IM due to a high incidence of PPH [39]. As the price differential between 5 IU/mL and 10 IU/mL ampoules is small [32], it is recommended that procurement focuses on 10 IU/mL ampoules to limit the possibility of sub-therapeutic dosing when used for PPH, while still providing adequate coverage for other indications.

### Regulatory issues

National medicines regulatory authorities are responsible for the maintenance of quality pharmaceuticals within the country, but with thousands of different medicines and entry points into the country, the regulatory task is challenging. WHO carried out a survey of over 100 peer-reviewed papers that reported on regulatory medicine testing in LMICs between 2007 and 2013 using non-randomized convenience sampling [31, 40]. Of the more than 48,000 samples tested, 10.5% failed across medical products [41]. Failure referred to inappropriate levels of API, but the acceptable range varied across the studies from 95 to 105% to 85–115% of expected amounts. This survey was not intended to provide a failure rate for medicine quality, but instead, to highlight the reality of substandard medicines in LMICs. A similar study surveyed over 400,000 medicine samples and found over 13% failed quality testing [40]. WHO’s recommended response for reducing this high failure rate is to prevent the manufacture, sale and consumption of substandard and falsified medicines; detect their presence in the supply chain; and respond quickly to incidents [41]. Although WHO and other organizations continue to strengthen the capacity of regulatory authorities, effective regulatory oversight is an on-going challenge, often due to technical capacity and resource constraints [42] In Africa, harmonization initiatives have helped to address these challenges through resource mobilization and by facilitating collaboration to enable regional reliance and work sharing to efficiently evaluate medicines.

#### Marketing authorization

Marketing authorizations are intended as a tool to ensure quality medicines and require a level of effort and expertise from the regulatory authorities. Before a medicine can be marketed and made available to patients, it must receive marketing authorization—often referred to as registration—by a Medicines Regulatory Authority (MRA). Marketing authorization applications for a multisource product, such as oxytocin injection, contain information to evaluate the safety, efficacy and quality of the product, and these applications require a certain level of expertise to properly evaluate. However, many LMICs have limited regulatory capacity, in both resources and expertise, to perform a rigorous regulatory evaluation to ensure the quality, safety and efficacy of pharmaceutical products that enter their markets [43]. To support stronger regulation in LMICs, joint dossier assessments have been completed through harmonization initiatives in the East African Community; unfortunately, oxytocin has not yet been approved via this mechanism [44]. Other regulatory mechanisms (e.g., WHO PQ Collaborative Registration Procedure [CRP] for Accelerated Registration, European Medicines Agency’s [EMA] Article 58 procedure, and SwissMedic’s Marketing Authorisation for Global Health Products [MAGHP]) have been created by the international community to contribute to public health and address resource and technical expertise challenges.

The regulatory processes can sometimes be time-consuming and extend the amount of time before quality products are available in the market. To improve the efficiency of regulatory reviews by national medicines regulatory authorities (NMRAs), WHO developed collaborative procedures for accelerated registration that allow countries to share registration information for WHO-prequalified and SRA-approved finished pharmaceutical products. These processes improve efficiency by reducing redundancy of reviews by. As of 2019, only two oxytocin FPPs were WHO-prequalified and eligible for accelerated registration and, to date, the process has not been used to register WHO-prequalified or SRA-approved oxytocin in a single country. Additionally, the EMA Article 58 procedure and MAGHP have not been used for oxytocin.

#### Market entry of unregistered oxytocin products

Product registration is an important tool for maintaining a quality supply of medicines throughout the country. However, there are two ways that unregistered oxytocin products enter a country—legally through a waiver process or illegally—both of which can contribute to the prevalence of poor-quality oxytocin in the market. The waiver process, typically used to quickly mitigate stockouts or to address new treatment standards, can be beneficial and help accelerate oxytocin procurements in the case of public health emergencies, but may also be problematic and allow product to enter the country without local evaluation for safety and efficacy and, perhaps, without NMRA involvement [45]. For instance, Indonesia has a special access scheme for medicines whereby the Ministry of Health issues the certificate of importation for unregistered medicines in collaboration with the NMRA. Other countries, such as Ghana, may not grant waivers for oxytocin procurement [46], but unregistered oxytocin products may still be prevalent. According to a 2013 study carried out in Ghana, 98% (*N* = 94) of oxytocin samples that did not pass quality testing were also not registered [15]. Pharmaceutical products that enter the market without registration, but instead through the waiver process, can undermine the regulatory process and lead to breaches in quality. However, the most significant threat to quality remains the illegal importation without knowledge of the regulatory authority.

#### National Medicines Regulatory Authority Capacity

NMRAs are responsible for ensuring that oxytocin products—either locally produced or imported—meet quality standards through rigorous evaluation. Evaluation occurs as part of a regulatory submission for marketing authorization and includes a critical review of stability data to confirm that oxytocin injection packaging and labeling have appropriate storage condition guidance.

One issue that limits quality assurance in LMIC regulatory systems is the ability of the regulator to ensure that products are appropriately labeled for use in the country. Because oxytocin is thermally labile, the conditions in which the stability studies are conducted should reflect local climatic conditions (see Distribution, transportation, and storage conditions), and LMIC regulatory authorities should not register products that have not been tested for the local conditions. Oxytocin products that are granted marketing authorization in LMICs may be those that were labelled for cooler, more temperate climates (i.e., labelled for non-refrigerated storage) and may not be stable when stored outside the cold chain in hotter climatic conditions.

A second issue that limits quality assurance in LMICs is whether the regulatory capacity can ensure that only quality products enter the market. In a survey of 26 countries in Sub-Saharan Africa, only 19 of 26 (73%) NMRAs included some evaluation of technical documents (e.g., manufacturer inspection reports or dossier materials) and, more generally, technical evaluation standards were not in compliance with WHO [43].

Finally, another issue that affects quality assurance is the requirement for on-going post-market surveillance which requires product testing. At upstream points in the supply chain, quality should be assessed using validated quality control test procedures; however, these tests are not practical at health facilities or hospitals as they require specialized equipment and technical expertise. Emerging technologies such as paper-based tests and point-of-use sample testing are being investigated [47, 48]. To date, there is no test to assess oxytocin content of the injection products at lower levels of the health system, highlighting the importance of on-going surveillance throughout the entire health supply chain.

## Recommendations

Specific available tools and approaches to ensure the quality of oxytocin are currently being implemented in several countries and are further described below. These recommendations are predicated on the consideration that there is an ethical duty to minimize avoidable risks due to poor-quality medicines [49].

### Promote good regulatory practices

National regulators must follow good regulatory practice including internationally recognized processes and procedures that can be used to improve the quality and cost-effectiveness of domestic regulations (see Regulatory issues). Increasingly, NMRAs must consider more robust models of regulation that account for resource constraints, increasingly complex technologies, globalization and public expectations. Regulators must implement risk-based review and oversight, including accelerated evaluation pathways or provisions to use expert recommendations such as those coming out of this evidence review. Specific to oxytocin injection, regulators must require compliance with GMP including aseptic processing since post-production sterilization is not possible for oxytocin.

### Facilitate NMRA approval of appropriate product labeling

NMRA approval of product must include the condition that labeling reflects local climatic conditions (see Manufacturer stability tests). In countries with hot and/or humid climates (zones III and IV), regulators should only approve oxytocin labels that state, “Store in a refrigerator (2°C to 8°C).” Storage conditions specified on medicine labels have a direct impact on quality. Therefore, the product labeling that regulators approve, or which manufacturers use for their product, must be adequate to support continued quality assurance and reduce the risk of treatment failure in each local market. In support of this specification, manufacturers registering oxytocin injection should provide stability data collected under the appropriate climatic zone parameters and using a validated stability-indicating method; both are standard requirements when establishing storage conditions for a medicine.

NMRAs should also ascertain whether a pharmacopeial method employed by a manufacturer has been sufficiently validated for the manufacturer’s oxytocin injection product. In cases where manufacturers request storage conditions other than at 2-8 °C, as a minimum, the request must be supported with data that relates to the climatic conditions of the procuring country. However, given the currently available evidence on the high prevalence of poor oxytocin injection quality and the likelihood for high temperature exposure during supply and storage in many LMICs, it is recommended that NMRAs require storage of all oxytocin injection products between 2 and 8 °C. Some stakeholders in LMICs have taken steps in line with this recommendation. In Kenya, the Ministry of Health recognized the WHO/UNICEF recommendation [50] around refrigeration of oxytocin (see Integrate oxytocin into existing health cold chains, as appropriate) and the government has excluded all oxytocin suppliers with products labelled for storage outside of the cold chain from national tenders [51].

### Implement ongoing post-market product quality surveillance

Implement post-market surveillance for oxytocin quality throughout the supply chain and close to the point of use (e.g., at the hospital storage facility). This task is difficult near the point-of-use since there are no simple rapid screening technologies that will allow a rapid analysis before administering oxytocin to the patient (see National Medicines Regulatory Authority Capacity). However, in Table 1, the inclusion of several PMS studies with more frequent occurrences may indicate that surveillance is increasingly becoming a priority. As previously discussed, ensuring procurement of quality oxytocin is critical, but may not be enough, to ensure quality at the end use. Since oxytocin degrades at elevated temperatures, the detection of substandard oxytocin through on-going quality surveillance remains important along all points of the supply chain.

### Procure quality-assured, appropriately labeled oxytocin products

Procurement agents at all levels of the health supply chain should procure quality-assured and appropriately labelled oxytocin in 10 IU ampoules to reduce confusion on storage and use of oxytocin (see Recommended dose). National and sub-national policies must prioritize procurement of high-quality oxytocin and require its appropriate storage and management. Procurement practices must ensure that quality is always retained even when there are pressures to select lower-priced oxytocin products.

As described in the *Model Quality Assurance System* for procurement agencies and similar procurement guidelines, “Procurement should be done with the aim of purchasing effective, quality assured products, and should not be focused on price alone [52].” Given the thermal-labile nature of oxytocin injection, quality assurance as part of the procurement process should include: the selection of registered oxytocin injection with storage conditions between 2-8^o^ C that is preferably WHO prequalified or approved by an SRA; and the requirement that appropriate temperatures are maintained during shipment and storage, throughout the supply chain. While this may appear as a challenge for low-resource countries, the PMS study in Ethiopia (Table 1) showed that procurement of quality-assured oxytocin coupled with appropriate storage and management is possible in these settings, even with budgetary and resource constraints [9]. Furthermore, the presence of oxytocin products within specification indicate potential positive effects on product quality [9] and make a case for strong political-will to actualize this recommendation.

### Integrate oxytocin into existing health cold chains, as appropriate

Store oxytocin from 2 to 8 °C throughout the supply chain to maintain its quality. While short temperature excursions may not harm product quality, cumulative heat exposure is generally not tracked.

The provision of consistent guidance on maintaining oxytocin at 2–8 °C during distribution and storage at warehouses or in facilities along the supply chain is important for maintaining quality. Where cold chain infrastructure is lacking for essential medicines, oxytocin could be included in existing functional cold chains, such as those used for vaccines, in locations where the supply chains overlap. WHO and UNICEF issued a joint statement encouraging countries to leverage cold chain resources, such as the Expanded Program on Immunization (EPI) cold chain, to ensure appropriate management of other temperature-sensitive products such as oxytocin [50]. Since the issuance of the joint statement, several countries have considered the options for integration into the cold chain [53, 54]. In 2017, the government of Uganda issued an official statement mandating integration of oxytocin in the EPI cold chain [55]. The EPI cold chain is tailored to meet the needs of vaccinations, often maintaining storage at mid-level warehouses and using short-term cold boxes for campaigns. EPI warehouse storage refrigerators may be useful for oxytocin, but short-term transportation to village health campaigns is probably not useful for oxytocin. The specific conditions where the EPI cold chain will be useful for oxytocin will vary depending upon the country context and a detailed understanding of the EPI cold chain [56].

### Consider adapting and using time-temperature indicators

Consider whether oxytocin may benefit from the use of time-temperature indicators (TTIs) on packaging as a mechanism to know when the ampoule’s heat exposure will have likely degraded the oxytocin. For other heat-sensitive products, specifically vaccines, TTIs have been used to monitor temperature exposure of a product over time and indicate when the product has reached its time-temperature end point [57]. A similar approach has been proposed for oxytocin, but so far, without evidence to link the use of TTIs to improved quality of oxytocin at the end user or improved health outcomes [58]. There have been two cases in Ghana where TTIs were used for oxytocin. In the first case, TTIs were affixed to oxytocin in a single-use injection device (Uniject) as part of a study of community-based distribution [59]. TTIs were used to signal the need to discard the product due to temperature excursions experienced under normal distribution conditions. In a second case in Ghana, TTIs were used to monitor heat exposure in selected facilities to determine the feasibility of distributing oxytocin through the public supply chain [60]. In the study, 1800 TTIs were affixed to 1800 boxes of 10 IU ampoules of oxytocin at the central level, which were then distributed to lower-level warehouses and facilities [60].

Both experiences suggest that use of TTIs could be used as part of a strategy for assuring oxytocin quality, but additional considerations would need to be reviewed. If taken to scale, TTIs should be affixed at the point of manufacture to ensure that time and heat exposure are accounted for during warehousing and transportation prior to arrival at a national central warehouse. Although existing TTIs are not specifically designed to monitor oxytocin stability, calibration to the thermal degradation of oxytocin is likely possible. On-going concerns about using TTIs include cost-benefit analysis and whether LMIC regulatory authorities would ensure that non-conforming oxytocin would not compete with more expensive TTI-compliant oxytocin.

## Conclusion

Key actions for regulatory agencies and supply chain systems are described that would reduce the presence of poor-quality oxytocin in LMICs (see High prevalence of poor-quality oxytocin in LMICs). Key actions include appropriate procurement practices, strong regulatory authority, appropriate labeling, cold chain resources in the supply chain. The procurement process—at the international, national or subnational levels—must include quality requirements for oxytocin injection. NMRAs can limit product registrations to include only oxytocin with, “Store in a refrigerator (2°C to 8°C)”, on the label. This consistent labeling will reduce incorrect oxytocin storage and will ensure that a single message about cold chain storage is clear throughout the supply chain. Supply chain systems can leverage existing cold chain resources—such as those used for vaccines—to protect oxytocin throughout transportation, storage and distribution processes, as appropriate. The maternal health community can urge donors, national governments, supply chain managers, maternal health programs and health providers to intensify stewardship and accountability.

## Data Availability

Not applicable.
